# Optimization of antimicrobial peptides for the application against biocorrosive bacteria

**DOI:** 10.1007/s00253-023-12562-9

**Published:** 2023-05-08

**Authors:** L. Stillger, L. Viau, L. Kamm, D. Holtmann, D. Müller

**Affiliations:** 1 Institute of Bioprocess Engineering and Pharmaceutical Technology, University of Applied Sciences Mittelhessen, Wiesenstrasse 14, 35390 Giessen, Germany; 2 Institute of Pharmaceutical Technology and Biopharmacy, Philipps-University Marburg, Biegenstraße 10, 35307 Marburg, Germany

**Keywords:** Microbiologically influenced corrosion, MIC, Lipidation, Amidation, Peptide stability

## Abstract

**Abstract:**

Microbiologically influenced corrosion is a common problem in the industrial field due to the deterioration of metals in the presence of various microorganisms, in particular sulfate-reducing bacteria (SRB) and sulfur-oxidizing bacteria (SOB). A common method to reduce microbiologically influenced corrosion is the application of biocides. The limited number of suitable biocides and the resulting development of resistance, high dosage, and high application rate hinder an effective application. An environmentally friendly alternative could be the application of antimicrobial peptides (AMP), which have already been established in the field of medical devices for a while. Here, the successful treatment of different AMPs against 3 SRB and 1 SOB was demonstrated. The peptide L5K5W was favored due to its broad activity, high stability, and simple structure resulting in low synthesis costs. An alanine scan showed that substitution of leucine with tryptophan increased the activity of this peptide twofold compared to the original peptide against *D. vulgaris*, the main representative of SRB. Additional optimization of this modified peptide through changes in amino acid composition and lipidations significantly increased the effectiveness, finally resulting in a minimum inhibitory concentration (MIC) of 15.63 μg/mL against *Desulfovibrio vulgaris*. Even against the marine SRB Desulfovibrio indonesiensis with a required salt concentration of min. 2%, an activity of the peptides can be observed (MIC: 31.25 μg/mL). The peptides also remained stable and active for 7 days in the supernatant of the bacterial culture.

**Key points:**

*• Antimicrobial peptides provide an alternative to combat biocorrosive bacteria.*

*• Optimization of the peptide sequence leads to a significant increase in activity.*

*• The investigated peptides exhibit high stability, both in the medium and in the bacterial supernatant.*

**Graphical abstract:**

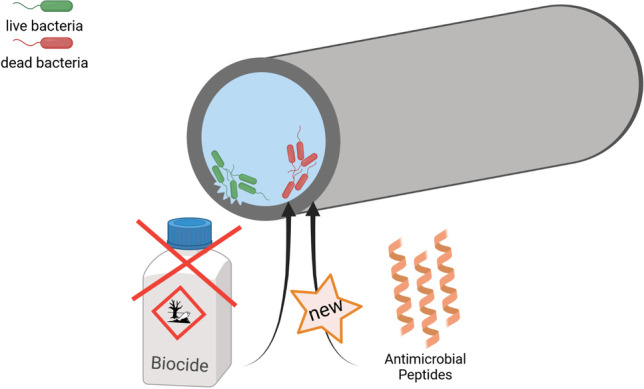

**Supplementary Information:**

The online version contains supplementary material available at 10.1007/s00253-023-12562-9.

## Introduction

Microbiologically influenced corrosion is a common problem due to the damage of metals in the presence of various microorganisms, such as anaerobic sulfate-reducing bacteria (SRB) like *Desulfovibrio* spp. and aerobic sulfur-oxidizing bacteria (SOB) like *Thiomonas* spp. (Dou et al. 2021). Thereby, the SRB have a crucial role in the formation of biocorrosion. The activity of the SRBs leads to the reduction of sulfate to hydrogen sulfide via the utilization of organic compounds or hydrogen as electron donors (chemical-microbial corrosion). Another mechanism is the removal of electrons from metallic compounds such as ferrous materials, which is required for the production of hydrogen sulfide and their further reaction to form iron sulfide (electrical-microbial corrosion) (Enning und Garrelfs 2014). The combination of the two bacterial groups (SRB + SOB), especially at changing oxygen levels through fluctuations in the water height, leads to immense damages. At low water levels, oxidation of the iron sulfides takes place and they are further oxidized to sulfuric acid by the SOB in the presence of oxygen (acid corrosion). However, at high water level, the oxygen content is limited and the SRBs can reduce the sulfate again (Barton and Fauque 2009).

The current method of treatment involves the use of biocides. However, the disadvantage here is the rapid resistance formation. This leads to the necessity to apply higher and higher concentrations in ever shorter time intervals. This is mainly enhanced through the low number of suitable biocides, with tetrakis(hydroxymethyl)phosphonium sulfate (THPS) and glutaraldehyde being the main ones (Jia et al. 2019a). Recent efforts to reduce the biocide concentration through additives have already been successfully demonstrated against SRB. By adding a mix of 8 different D-amino acids to THPS, the obtained effect was comparable to the double concentration of THPS alone (Jia et al. 2017). Identical result could be achieved with the addition of a cyclic peptide (Jia et al. 2019b). The addition of chelates can also significantly improve the effect of the biocide glutaraldehyde against two SRB, *D. desulfuricans* (Wen et al. 2009) and *D. vulgaris* (Wen et al. 2012). Further researches are focusing on herbal additives such as D-limonene, which also demonstrates an enhanced effect in combination with glutaraldehyde (Kijkla et al. 2021). The problem with all these modifications is that the additives alone have no antimicrobial properties. Thus, biocide application is still necessary.

An alternative treatment strategy could be the use of antimicrobial peptides (AMP). These naturally occurring peptides have already demonstrated their antimicrobial impact in medical applications (Lin et al. 2018). Specific applications of AMPs against biocorrosive bacteria, specifically SRB or SOB, are rarely described. Initial experiments were performed by expressing the AMP in a host organism (*Bacillus subtilis* BE1500) to circumvent the diffusion processes into the SRB biofilm. The disadvantage of this method was that the AMPs also attacked the host organism and thus reducing the applicability of the in situ production of AMPs. Furthermore, the application of a genetically modified organism will be only possible in a limited number of applications. Nevertheless, the AMPs of this study showed antimicrobial property against *D. vulgaris* and *D. gigas* (Jayaraman et al. 1999).

The aim of this study is to use the antimicrobial properties of AMPs to transfer the application of AMPs to the industrial sector and thus demonstrate an alternative to biocides. In this regard, this paper includes preliminary experiments in form of determination of minimum inhibitory concentration on biocorrosion bacteria (SRB: *D. vulgaris*, *D. desulfuricans*, *D. indonesiensis* and SOB: *T. intermedia*) and stability studies to demonstrate the potential of AMPs against biocorrosion bacteria.

## Materials and methods

### Peptide sequence selection

Based on two existing peptide databases, BaAMPs (Di Luca et al. 2015) and APD3 (Wang et al. 2016), suitable AMP sequences with the following characteristics were searched: positive charge of approx. +5, amphiphilicity as high as possible, hydrophobicity around 50%, sequences as short and simple as possible. Thereby, suitable sequences were identified, prioritized according to the above criteria, and were synthesized and tested.

### Peptide synthesis

All peptides were synthesized by solid-phase in 0.1 mM scale with 9-Fluorenylmethoxycarbonyl (fmoc)-chemistry using microwave-assisted peptide synthesizer Liberty Blue™ (CEM GmbH, Kamp-Lintfort, Germany). Solvents were purchased from Carl Roth GmbH & Co.KG (Karlsruhe, Germany), amino acids and resins from Merck KGaA (Darmstadt, Germany) and Iris Biotech GmbH (Marktredwitz, Germany). First, swelling of the resin (preloaded or rink-amid) in 10 mL dimethylformamide (DMF) for 300 s was performed. The following steps were performed cyclically for each amino acid. First, deprotection with 3 mL of 20% piperidine in DMF, 90 °C, 65 s, 30 W followed by a washing step with 4 mL of DMF 3 times, and repeating these two steps. Then, coupling of 2.5 mL of 0.2 M amino acid at 90 °C, 125 s with 1 mL of N,N′-diisopropylcarbodiimide (0.5 M in DMF) and 0.5 mL of Oxyma Pure (1 M in DMF) and washing 2 times. Afterwards, final deprotection using 4 mL of 20% piperidine in DMF, 90 °C, 65 s followed by washing 3 times with 3 mL of DMF each. These last two steps were repeated twice. For the amino acid arginine, coupling was first performed at 25 °C,1500 s and then at 75 °C, 120 s, 30 W in duplicate, interrupted by one washing step. For the coupling of lipids, the normal coupling protocol of amino acids was applied (fmoc-6-aminohexan acid and fmoc-8-aminooctane acid), and if the lipids were not fmoc-protected (C2, C10, C12, C14), it was performed without final deprotection. After synthesis, the peptide was removed from the device and rinsed twice with DMF and 3× with dichloromethane followed by overnight lyophilization. The cleavage from the resin was incubated with 4 mL solution consisting of 85% trifluoroacetic acid, 5% triisopropylsilane, 5% 2,2′-(ethylenedioxy)diethanethiol, and 5% milliQ water (v/v/v/v) at room temperature for 3 h at 300 rpm. Filtration was used to separate the resin from the peptide. Precipitation of the peptide was performed with 5-fold volume of cleavage solution with ice-cold diethyl ether followed by centrifugation for 5 min at 5000 rpm (2×). Subsequently, the peptide pellet was dried under nitrogen for approx. 5 min and lyophilized using freeze-drying. An overview of the individual steps of the peptide synthesis can be observed in the Supplementary Information (Fig. S1).

The analysis of the peptides was performed on a RP-HPLC (Agilent 1100, detector: diode array detector G1315A, column: Phenomenex®-Aeris™ 2.6 μm Peptide XB-C18 100 Å,150 × 2.1 mm), and ESI-LC-MS was performed (Knauer, detector: Surveyor MSQ, column: Eurospher II 100-2 C18A, 100 × 2 mm, 2 μm). The gradient was performed with a flow rate of 0.25 mL/min from 99% of mobile phase A (0.1% TFA in milliQ) over 15 min to 95% of mobile phase B (0.1% TFA in acetonitrile), kept at 95% with B for 2 min, and equilibrated at 99% with A for another 3 min. 20 μL (RP-HPLC) and 10 μL (ESI-LC-MS) peptide solution (250 μg/mL in milliQ) was injected.

### Alanine scan and peptide optimization

For the alanine scan, each amino acid of L5K5W was replaced with alanine individually. The peptides obtained were termed L5K5W-A1 to A11. In addition, both positions 4 and 6 in L5K5W were replaced with the amino acid alanine (L5K5W-A4I6) as a double alanine scan and with the amino acid tryptophan (L5K5W-W4I6).

Rink amide resins were used for amidation of the peptides. Lipidation followed the identical coupling protocol for amino acids.

### Strains and cultivation

The following SRB were used: *Desulfovibrio vulgaris* DSM 644, *Desulfovibrio desulfuricans* DSM 642, and *Desulfovibrio indonesiensis* DSM 15121. The Postgate medium (DSMZ medium 63) with a modification of 0.004 g/L FeSO_4_ × 7 H_2_O and 0.3 g/L Tri-sodium citrate (and 2% NaCl for *D. indonesiensis*) was flushed with nitrogen gas. Each culture was subcultured 1:100. Cultivation occurred for 48 h, 37 °C (30 °C for *D. desulfuricans*), without shaking. The following SOB was used: *Thiomonas intermedia* DSM 18155. Therefore, the *Thiomonas* medium (DSMZ medium 35a) was used, subcultured 1:100, and cultivated for 48 h, 30 °C, 115 rpm. *Escherichia coli* K12 ATCC 25404 was grown in LB Lennox medium (Carl Roth GmbH & Co.KG Karlsruhe, Germany) for 18 h, 37 °C, 115 rpm and diluted 1:1000 in Mueller-Hinton broth (Carl Roth GmbH & Co.KG Karlsruhe, Germany). Incubation during the assay took place for 24 h, 37 °C, 115 rpm.

### Minimal inhibitory concentration (MIC) assay

A stock solution of 1 mg/mL in milliQ from the respective peptide and a concentration range of 1000–1.95 μg/mL was prepared by serial 2-fold dilution. 20 μL of each test substance (*n*=3) per well was added to a 96-well microtiter plate (polystyrene). 100 μL of each bacteria suspension (start OD600: 0.001) was added to the well with test substance, as well as 120 μL as growth control (*n*=35) and 120 μL medium (*n*=1) as sterile control to an empty well. To avoid evaporation, all microtiter plates were cultivated in air-tight containers during the incubation period. Microtiter plates for testing on SRB were prepared in the anaerobic chamber (Coy Laboratory Products Inc. Grass Lake, United States of America) and cultivated in air-tight containers including Oxoid anaerobic bag (Thermo Fisher Scientific Inc. Waltham, United States of America) to ensure anaerobic conditions. The microtiter plates were measured before and after incubation at OD600, and the growth inhibition in % was determined with the following equation:

1 $$\textrm{inhibition}\ \left[\%\right]=\frac{\Delta \textrm{OD}{600}_{\textrm{test}\ \textrm{substance}}}{\Delta \textrm{OD}{600}_{\textrm{growth}\ \textrm{controle}}}\bullet 100\%$$

The minimum inhibitory concentration (MIC) describes the lowest concentration where no bacterial growth could be detected. Besides the MIC100, where a complete reduction of bacterial growth could be detected, other partial inhibitions such as MIC95 or MIC50, where 95% or 50% growth inhibition occurs, can be specified.

### Stability tests

For the stability tests, a bacterial suspension (7 days old) of *D. vulgaris* was centrifuged at 5000 rpm for 10 min. 1 mg/mL peptide solution was prepared within the supernatant and incubated at 37 °C for 7 days. The respective aliquots (*t*=0, 48, 72, 168 h) were mixed 1:10 with quenching solution (milliQ + 0.08% trifluoroacetic acid) and analyzed in triplicates according to the protocol described above for RP-HPLC. In addition, after incubation in the supernatant (168 h), the peptide (P3.2.) was analyzed for activity using the MIC assay.

## Results

### Antimicrobial peptides against SRB and SOB

A MIC95 of 62.5 μg/mL was achieved with 2 AMPs against *D. vulgaris* (S6L3-33 and bactenecin). Although high salinity results in a significant reduction or even complete loss of effectiveness of AMPs (Löfgren et al. 2009), a considerable activity (31.25 μg/mL with bactenecin) against the marine bacterium *D. indonesiensis* was detected despite 2% NaCl in the culture medium. An activity can also be determined for two other peptides (S6L3-33 and DASamP1) despite a salt content of 2%. Since seawater has an average salt content of around 3.5%, the activity of peptide S6L3-33 was also tested exemplary. The MIC95 in this case is 500 μg/mL, indicating that the maintenance of peptide activity at high salinity is reduced but still possible. For the bacteria, *D. desulfuricans* and *T. intermedia*, it was difficult to achieve a complete inhibition with the tested AMPs. Therefore, the MIC50 was considered. Here, the peptides S6L3-33 and bactenecin showed the best effectiveness (Table 1).

**Table 1:** MIC (μg/mL) at 95% (MIC95) or 50% (MIC50) inhibition for 3 SRB (*D. vulgaris*, *D. desulfuricans*, and *D. indonesiensis*) and 1 SOB (*T. intermedia*) for the 4 preselected peptides; incubation: 48 h, 37 °C (*D. vulgaris + D. indonesiensis*) or 30 °C (*D. desulfuricans, T. intermedia*), without shaking (SRB) or 115 rpm (SOB)

		*D. vulgaris*	*D. desulfuricans*	*D. indonesiensis*	*T. intermedia*
Peptide name	Sequence	MIC95	MIC50	MIC95	MIC50
L5K5W	KKLLKWLKKLL	125	>1000	>1000	500
S6L3-33	FKKFWKWFRRF	62.5	500	125	62.5
Bactenecin	RLCRIVVIRVCR	62.5	250	31.25	62.5
DASamP1	FFGKVLKLIRKIF	125	500	500	125

Since *D. vulgaris* is considered a model organism for SRB and is frequently used in experiments with SRB (Heidelberg et al. 2004), the selection of the best peptide is primarily based on its effectiveness against this bacterium. In addition, stability and simplicity of the sequence with a minimum number of different amino acids and the simplest possible amino acids to reduce synthesis costs were crucial. Based on these criteria, L5K5W was selected for further optimization.

### Alanine scan of L5K5W

**Fig. 1:**
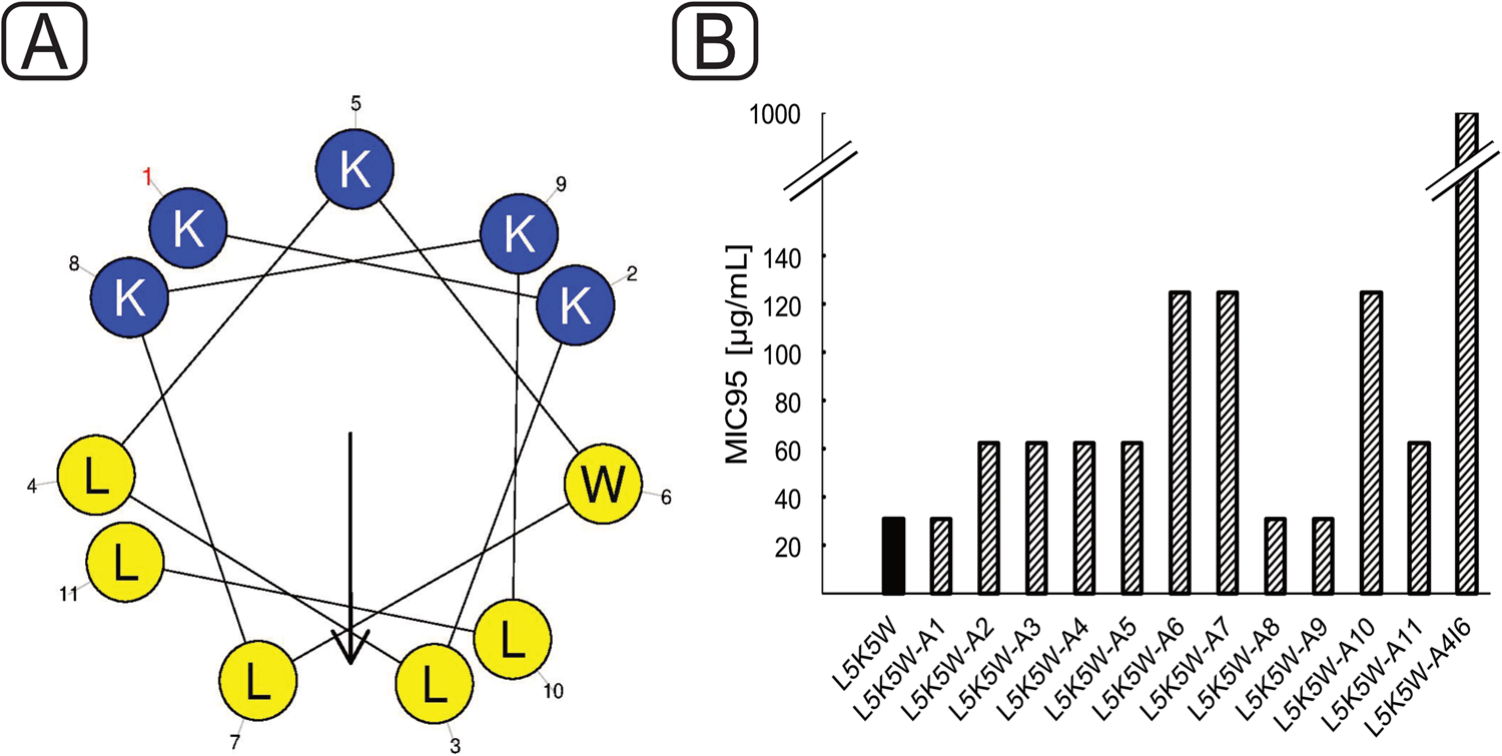
Helical structure of L5K5W (**A**), simulated with HeliQuest (Gautier et al. 2008); MIC95 (μg/mL) of the original peptide L5K5W (black), 11 peptides of the alanine scan L5K5W-A1 to L5K5W-A11 and peptide L5K5W-A4I6 of the double alanine scan (striped), tested against *E. coli* K12, incubation: 24 h, 37 °C, 115 rpm (**B**)

The helical structure of the peptide is shown in Fig. 1A. Replacement of the amino acid (Fig. 1B) at positions 6, 7, and 10 results in an increase in the required inhibitory concentration from 31.25 μg/mL to 125 μg/mL. The exchange at positions 2–5 and 11 causes a reduction of the activity up to a MIC95 of 62.5 μg/mL, while the exchange at positions 1, 8, and 9 did not lead to any loss of activity. A double alanine scan was performed (L5K5W-A4I6) where the amino acid alanine was inserted at both positions 4 and 6. This resulted in an immense loss of activity (MIC95: 1000 μg/mL). In addition to position 6, tryptophan was also inserted at position 4, reducing the MIC95 against *D. vulgaris* from 125 μg/mL to 62.5 μg/mL (Table 2).

**Table 2:** MIC (μg/mL) at 95% (MIC95) or 50% (MIC50) inhibition for 3 SRB (*D. vulgaris*, *D. desulfuricans*, and *D. indonesiensis*) for L5K5W and L5K5W-W4I6 (modification colored in blue) and its modified peptides (modifications colored in red), amino acids in 1-letter code; incubation: 48 h, 37 °C (*D. vulgaris + D. indonesiensis*) or 30 °C (*D. desulfuricans*), without shaking

		*D. vulgaris*	*D. desulfuricans*	*D. indonesiensis*
Peptide name	Sequence	MIC95	MIC50	MIC95
L5K5W	KKLLKWLKKLL	125	>1000	>1000
Modification of amino acid composition after alanine scan				
L5K5W-W4I6	KKLWKWLKKLL	62.5	>1000	>1000
Modification of amino acid composition				
P1.1.	RRLWRWLRRLL	62.5	1000	250
Modification of the C-terminus				
P2.1.	KKLWKWLKKLL-NH_2_	31.25	250	125
Modification of the N- and C-terminus				
P3.1.	C2-KKLWKWLKKLL-NH_2_	31.25	500	125
P3.2.	C6-KKLWKWLKKLL-NH_2_*	15.63	250	125
P3.3.	C8-KKLWKWLKKLL-NH_2_*	15.63	250	62.5
P3.4.	C10-KKLWKWLKKLL-NH_2_	31.25	125	31.25
P3.5.	C12-KKLWKWLKKLL-NH_2_	62.5	62.5	31.25
P3.6.	C14-KKLWKWLKKLL-NH_2_	125	125	62.5

*Amidated lipid

### Optimization of L5K5W-W4I6

Since SRBs are considered to initiate biocorrosion through their reduction of sulfur compounds, this paper will henceforward only focus on these bacteria.

The peptide L5K5W-W4I6 that emerged as a result of the alanine scan was first modified by further changes in its amino acid composition (Table 2). For this purpose, lysine was replaced by arginine (P1.1.). In this case, an increased activity could be observed against *D. desulfuricans* and *D. indonesiensis*, while the same MIC95 could be detected in comparison to the same peptide with lysine (L5K5W-W4I6) against *D. vulgaris*. In addition, the respective termini of the peptide were modified. Amidation of the C-terminus of the peptide (P2.1.) results in an increased activity for all 3 SRB representatives studied here. The coupling of lipids of different lengths (C2, C6, C8, C10, C12, and C14) to the N-terminus of the peptide results in an increased activity. By modifying the C- or N-terminus, an MIC of 15.63 μg/mL against *D. vulgaris* can be achieved, which is 2 dilution steps lower than for the original sequence. However, the enhancement of this modification was more crucial for the other SRB, where the lowest MIC of 62.5 μg/mL (*D. desulfuricans*) and 31.25 μg/mL (*D. indonesiensis*), respectively, could be achieved while no antibacterial effect could be detected with the original sequence. The lowest MIC against *D. vulgaris* was achieved with C6 and C8 (P3.2. and P3.3.), whereas longer lipids were required to achieve the lowest MIC for *D. desulfuricans* (P3.5.) and *D. indonesiensis* (P3.4. and P3.5.).

### Stability of peptides in culture supernatants

By directly measuring the peptide’s stability in the supernatant of a culture, the stability can be analyzed at identical conditions as during cultivation. This eliminates the necessity to prepare complicated mixtures of the respective proteases and thus represents a simple and more realistic method for stability analysis.

**Fig. 2:**
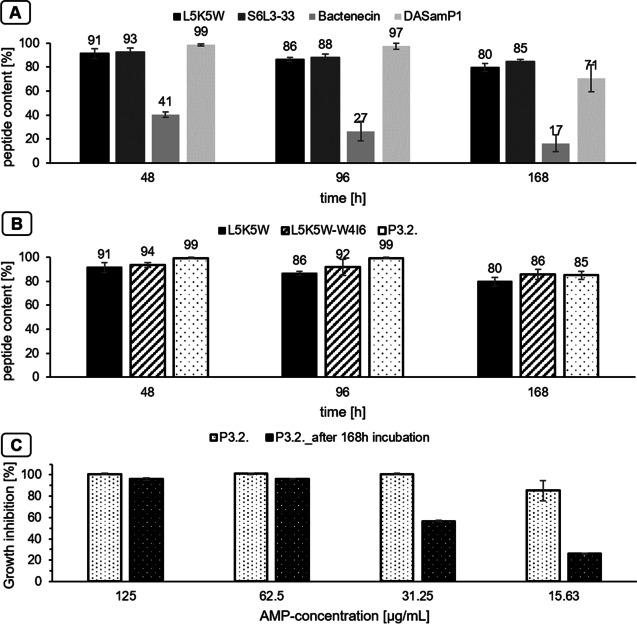
Stability in the supernatant of a 7-day-old culture of *D. vulgaris* of the 4 peptide favorites L5K5W (black), S6L3-33 (dark gray), bactenecin (gray), and DASamP1 (light gray) (**A**), of L5K5W (black) and its best modifications—L5K5W-W4I6 (striped) and P3.2. (dotted) (**B**), analyzed with RP-HPLC, 100% peptide content corresponds with the peptide content at 0 h; growth inhibition of *D. vulgaris* (%), incubation 48 h, 37 °C without shaking for different AMP concentrations (μg/mL) of P3.2. (dotted white) and P3.2. after incubation for 168 h in the supernatant (dotted black) (**C**); values shown as means with standard deviation *n*=3

Thereby, the peptides L5K5W, S6L3-33, and DASamP1 demonstrated only a low degradation, and even after an incubation time of 168 h, a peptide content of more than 80% was still present for L5K5W and S6L3-33. Bactenecin had a significant stability loss after only 48 h (Fig. 2A). The optimization of the selected peptide L5K5W resulted on the one hand in an increased activity and on the other hand in an increased stability (Fig. 2B). A complete maintenance of stability for up to 96 h and afterwards only a low decrease could be detected, whereas nearly no peptide loss could be detected in stability studies in medium (Postgate C), with only exception of bactenecin (see Fig. S2). Not only the amount of the peptide is important but also the maintenance of the activity (Fig. 2C). Therefore, the peptide (P3.2.) was tested with the MIC assay against *D. vulgaris* after incubation (168 h) in the supernatant. For this purpose, the peptide (1 mg/mL) was first incubated in the supernatant for 168 h, wherefrom the dilution series for the MIC assay was subsequently prepared. The peptides continued to show a good activity with an MIC95 of 62.5 μg/mL and an MIC50 of 31.25 μg/mL. This was only slightly higher compared to direct testing without incubation.

## Discussion

The selection of AMPs focused on the membranolytic mode of action to address all the different species involved in biocorrosion. Potential membranolytic sequences should achieve the following criteria (Brogden 2005; Bahar and Ren 2013). Positively charged amino acids have to be present in the sequence to allow peptide interaction to the negatively charged bacterial membrane via electrostatic interactions. Even though the increase in positive charges leads to an increase in activity, it also damages eukaryotic cells through to pore formation (Jiang et al. 2008). Since environmental damage due to the application of AMPs is to be avoided, damage to eukaryotic cells should be excluded for the protection of animals and humans. Thus, a charge of approx. + 5 was set for these experiments as Tossi et al. declare an average charge between +4 and +6 as optimal (Tossi et al. 2000). Another parameter is given by the hydrophobicity of the peptide, which allows the peptide to be integrated into the lipid bilayer. However, if the hydrophobicity is high, the peptides penetrate deeper into the membrane, resulting in an increased cytotoxicity (Chen et al. 2007). Therefore, a hydrophobicity range of 40–60% is considered to be optimal (Tossi et al. 2000). An important trait is the strict separation of positive and hydrophobic amino acids in the peptide, the so-called amphiphilicity (Fernández-Vidal et al. 2007). The membranolytic effect initially aims at an electrostatic interaction of negatively charged peptide and positively charged bacterial membrane (Brogden 2005). For this purpose, a high amphiphilicity is crucial to generate the highest possible electrostatic interaction (Fernández-Vidal et al. 2007). This is followed by the integration of the peptide into the bacterial membrane due to hydrophobic interactions (barrel-stave and toroidal-pore model). For this purpose, a high amphiphilicity is also crucial, since the presence of the hydrophobic amino acids on one helix side allows the hydrophobic interactions to be maximal (Brogden 2005). The sequence should be kept as short as possible and consist of simple amino acids to reduce synthesis costs. In this case, the maximum number of amino acids was defined as 20. Based on these criteria, suitable sequences were chosen from official peptide databases, and in addition to a general database APD3 (Wang et al. 2016), one was also selected for its specifically antibiofilm-active sequences BaAMPs (Di Luca et al. 2015). Since biofilm occurs when SRB/SOB colonize metallic surfaces, these sequences could also be considered for further investigation against sessile bacteria.

The peptide sequences with the highest amphiphilicity primarily demonstrated the best effectiveness in the MIC assay against SOB and SRB. This is consistent with the conclusion of Fernández-Vidal et al. (Fernández-Vidal et al. 2007), who identified amphiphilicity as a crucial parameter for peptide structure. They analyzed peptides with the same amino acid composition and thus with the same hydrophobicity but different amphiphilicity. Thereby, a stronger helix formation was detected for peptides with a higher amphiphilicity, which is associated with an increased membrane effect (Fernández-Vidal et al. 2007).

The exchange at positions 6, 7, and 10 was classified as critical by the alanine scan. Assuming an alpha-helix structure of L5K5W, positions 7 and 10 are in the hydrophobic range (Fig. 1A) and a substitution significantly reduces the hydrophobicity and the hydrophobic moment of the peptide and thus the absorption of the peptide into the bacterial membrane. Tryptophan is described to be crucial for anchoring the peptide in the bacterial membrane, which is based on the ability of tryptophan during membrane interactions to stabilize the amphipathic properties of peptide helices (Won et al. 2006). That is the reason why tryptophan has a decisive role especially at the interface between the hydrophobic and hydrophilic regions (Kang et al. 2009). The alanine scan carried out here proves this, as a significant loss of activity can be seen when position 6 is replaced. To confirm the role of tryptophan at the amphiphilic interface, a double alanine scan was performed (L5K5W-A4I6) where the amino acid alanine was inserted at both positions 4 and 6 (MIC100: 1000 μg/mL against *E. coli*). In contrast, the double insertion of tryptophan at positions 4 and 6 (L5K5W-W4I6) reduced the required concentration to 62.5 μg/mL against *D. vulgaris.* This further confirms the enormous influence of tryptophan at this interface and provides the basis for the further optimization. The replacement of arginine by lysine often results in increased activity (Andreev et al. 2014). Arginine is the amino acid with the highest isoelectric point of 10.8 (Pu et al. 2013), which is the only amino acid that results in charge maintenance during interaction with the bacterial membrane (Li et al. 2013). Also, a higher number of hydrogen bond donors and a stronger atomic charge distribution of the guanidinium group in arginine instead of the amino group in lysine could be a reason, whereby according to Rice et al. the increase in activity is mainly based on the second point (Rice and Wereszczynski 2017; Meloni et al. 2020). Amidation of the C-terminus of the peptide (P2.1.) results in an addition of a positive charge, leading to improved binding to the bacterial membrane. This is reflected in the enhanced activity. Furthermore, coupling of lipids of different lengths (C2, C6, C8, C10, C12, and C14) to the N-terminus results in a significant increase in hydrophobicity, allowing the peptide to better integrate itself into the hydrophobic bacterial lipid bilayer (Ray et al. 2017). This is shown by a significant reduction of the MIC (Table 2). Since peptides with different lipid lengths demonstrated the best activity for each of the 3 SRBs, it was shown that not only the lipid length is crucial for lipidation but also the peptide sequence and the bacteria. Grimsey et al. confirmed this with respective modifications of two peptides on two gram-positive, *Staphylococcus aureus* and *Enterococcus faecalis*, and three gram-negative bacteria, *Salmonella typhimurium*, *Escherichia coli*, and *Pseudomonas aeruginosa* (Grimsey et al. 2020). Lipidation drastically increased the activity, especially against the marine bacterium *D. indonesiensis*, which can be attributed to the increased salt tolerance of the peptides due to lipidation (Krishnakumari and Nagaraj 2015).

The peptides L5K5W, S6L3-33, and DASamP1 can be considered as stable, and long-term applications with these peptides could be envisaged, especially as these peptides also exhibit nearly consistent stability in the medium (Fig. S2). In comparison to the other peptides, bactenecin is the only peptide that contains cysteines. These two cysteines can be oxidized to form a disulfide bridge, which stabilizes the peptide structure (Reddie and Carroll 2008). However, since *D. vulgaris* is cultivated in reduced medium, the cysteines are present in their reduced form with thiol groups. Since stability was not performed under anaerobic conditions, atmospheric oxygen may cause oxidation of the cysteines over time. Thus, the seeming loss of stability could also be caused by a change in the peptide conformation. This was consistent with the stability experiments of bactenecin in the medium, where it also resulted in peptide loss (Fig. S2). In particular, due to the lipidated N-terminus and the amidation at the C-terminus of the optimized peptides, the peptide ends are protected against exopeptidases, thus preventing degradation of the peptide by them. Both the high stability itself and the retention of the activity of the peptides allow long-term application.

The current strategy to control microbial-influenced corrosion is based on biocides, which lead to fast resistance, requiring higher and higher concentrations to eradicate the bacteria. Alternative treatment strategies are based on additives like D-amino acids or chelates to biocides to reduce the required biocide concentration by a synergistic effect. However, these additives are not inhibitory on their own, and although a reduced biocide concentration is required, the presence of biocide is still necessary (Jia et al. 2017; Wen et al. 2012; Wen et al. 2009). In this paper, a new method for the treatment of biocorrosion is described: the application of AMPs. It could be shown that peptide sequences from APD3 and BaAMPs databases were selected based on certain characteristics and successfully applied against biocorrosion bacteria. Optimization of the amino acid composition and modifications of the C- and N-terminus of the peptide resulted in a further significant increase in activity. Thus, a minimum inhibitory concentration (MIC) of 15.63 μg/mL was finally achieved against *D. vulgaris*. This provides an alternative to existing biocides such as THPS, which requires a concentration of 40 μg/mL (0.1 mM) to inhibit SRB growth (Greene et al. 2006). However, there exist some limitations in the peptide effect. Membranolytic AMPs have lower effectiveness in saline environments due to the interaction of salt ions with the charged peptide. This was observed for S6L3-33 against the marine SRB strain *D. indonesiensis*, where the MIC was significantly too high at a salinity of 3.5% to be established as a new strategy and is therefore not competitive to the existing biocide treatment. The stability studies showed that the peptides remained stable in the supernatant of *D. vulgaris*. However, since several bacterial species are involved in biocorrosion, or biocorrosion can occur in open systems such as the marine environment, numerous proteases can act with the peptides. Thus, it may lead to an early peptide degradation without any peptide action having occurred. In this context, the necessary surface immobilization due to open systems of AMPs should be mentioned, which, however, is a challenge due to the lack of functional groups of the metals and the guarantee of the mobility and the associated activity of the AMPs (Stillger and Müller 2022).

Even though this paper presents a novel approach to combating biocorrosion bacteria, this paper represents only the initial research and multiple investigations are still needed to establish AMPs as a protection method against biocorrosion. Nevertheless, this paper demonstrated an alternative treatment in the form of AMPs against biocorrosion bacteria using SRB and SOB.

## Supplementary information

ESM 1

## Data Availability

The data generated during and/or analyzed during the current study are available from the corresponding author on reasonable request.
