# Supplementary material for: Optimization of antimicrobial peptides for the application against biocorrosive bacteria
Source: Appl Microbiol Biotechnol. 2023 May 8;107(12):4041–9. doi: 10.1007/s00253-023-12562-9 (PMC10238325; doi:10.1007/s00253-023-12562-9)
Supplement: Supplementary file 1 — ESM 1 [file 253_2023_12562_MOESM1_ESM.pdf]

Journal of Applied Microbiology and Biotechnology

Optimization of antimicrobial peptides for the application against biocorrosive bacteria

Stillger, L.[1], Viau, L.[1], Kamm, L.[1], Holtmann, D.[1], Müller, D.[2*]

[1] Institute of Bioprocess Engineering and Pharmaceutical Technology, University of Applied Sciences Mittelhessen, Wiesenstrasse 14 35390 Giessen, Germany

[2] Institute of Pharmaceutical Technology and Biopharmacy, Philipps-University Marburg, Biegenstraße 10 35307 Marburg, Germany

* Correspondence: daniela.mueller@pharmazie.uni-marburg.de, telephone number +496421 28-25882, fax number +49 6421 28-27016

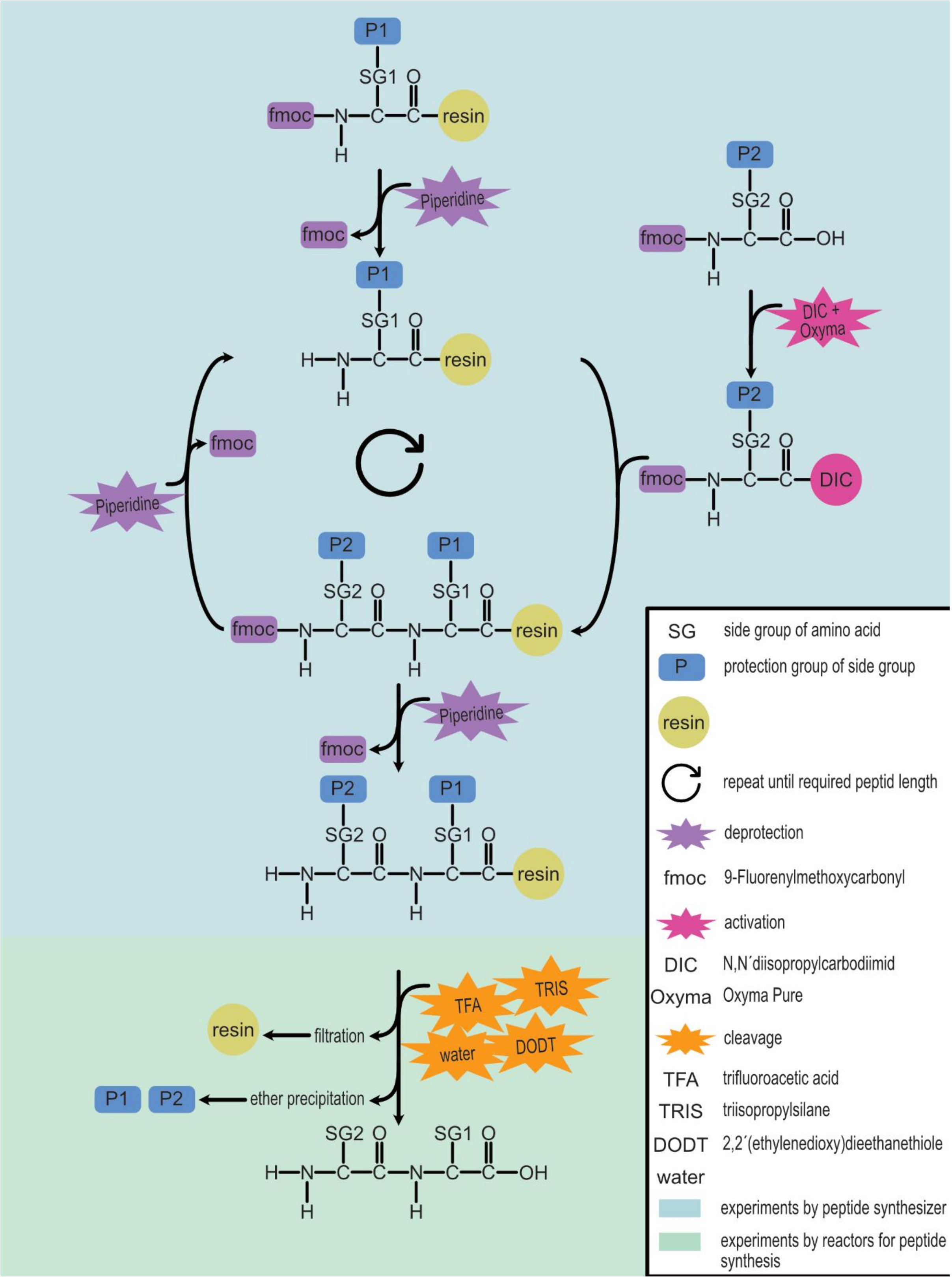

Fig. S1 Overview of the individual steps of the solid-phase peptide synthesis (SPPS)

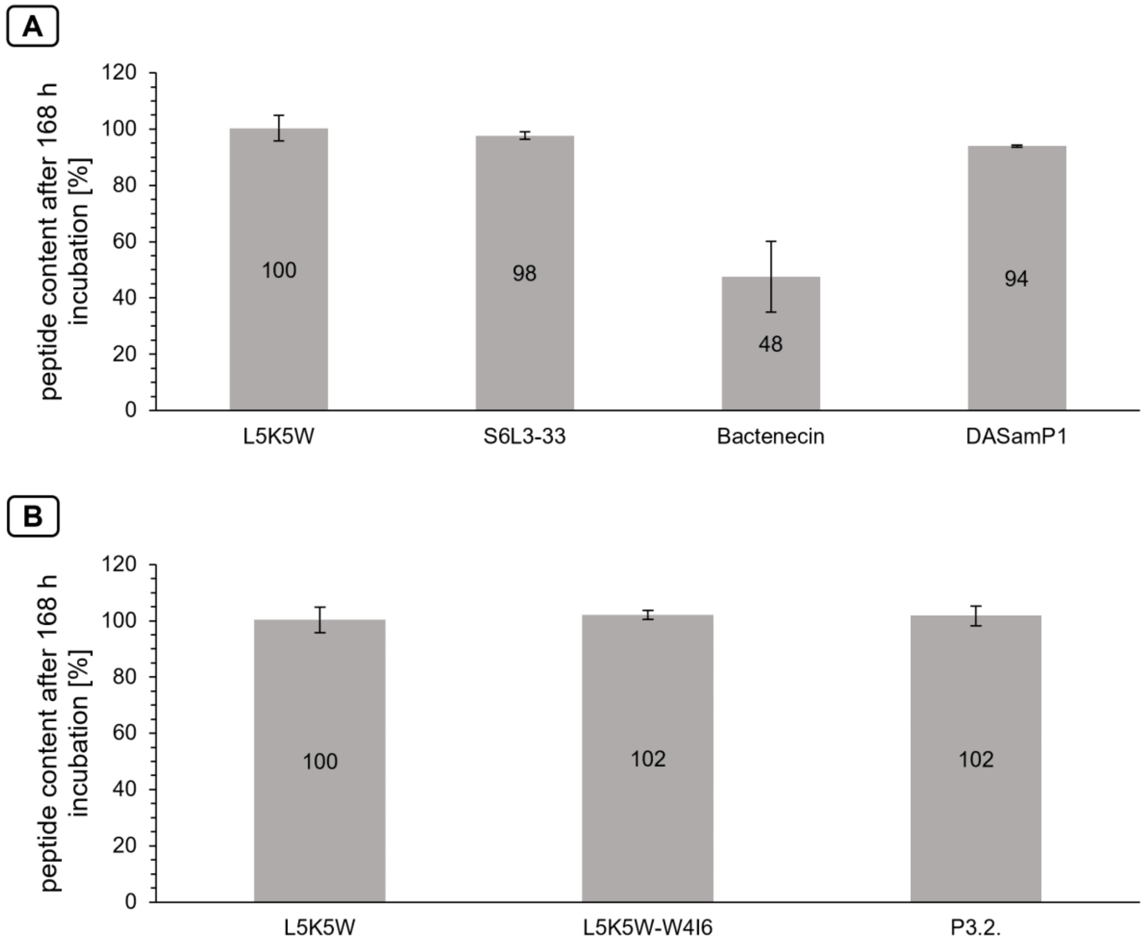

Fig. S2 Stability in cultivation medium (Postgate C) for *D. vulgaris* of the 4 peptide-favorites L5K5W, S6L3-33, Bactenecin and DASamP1 (A), of L5K5W and its best modifications – L5K5W-W4I6 and P3.2. (B) after 168 h incubation, analyzed with RP-HPLC, 100 % peptide content corresponds with the peptide content at 0 h; values shown as means with standard deviation n=3
